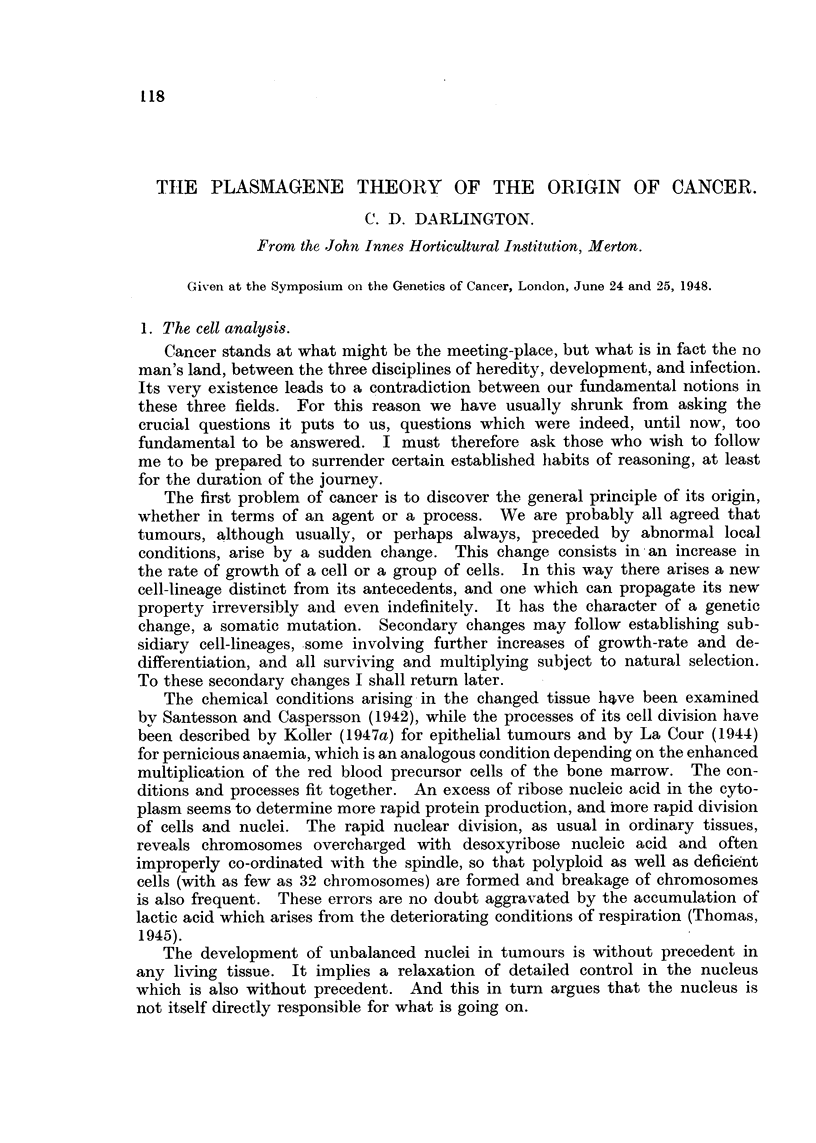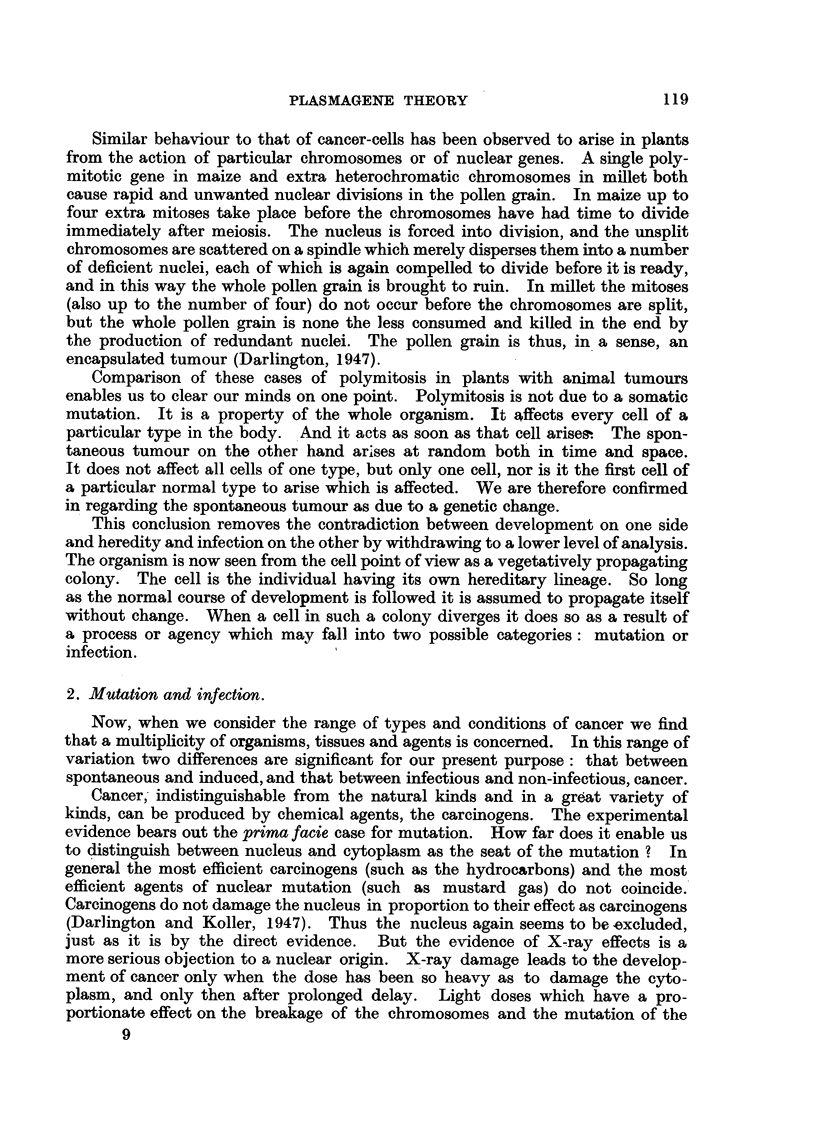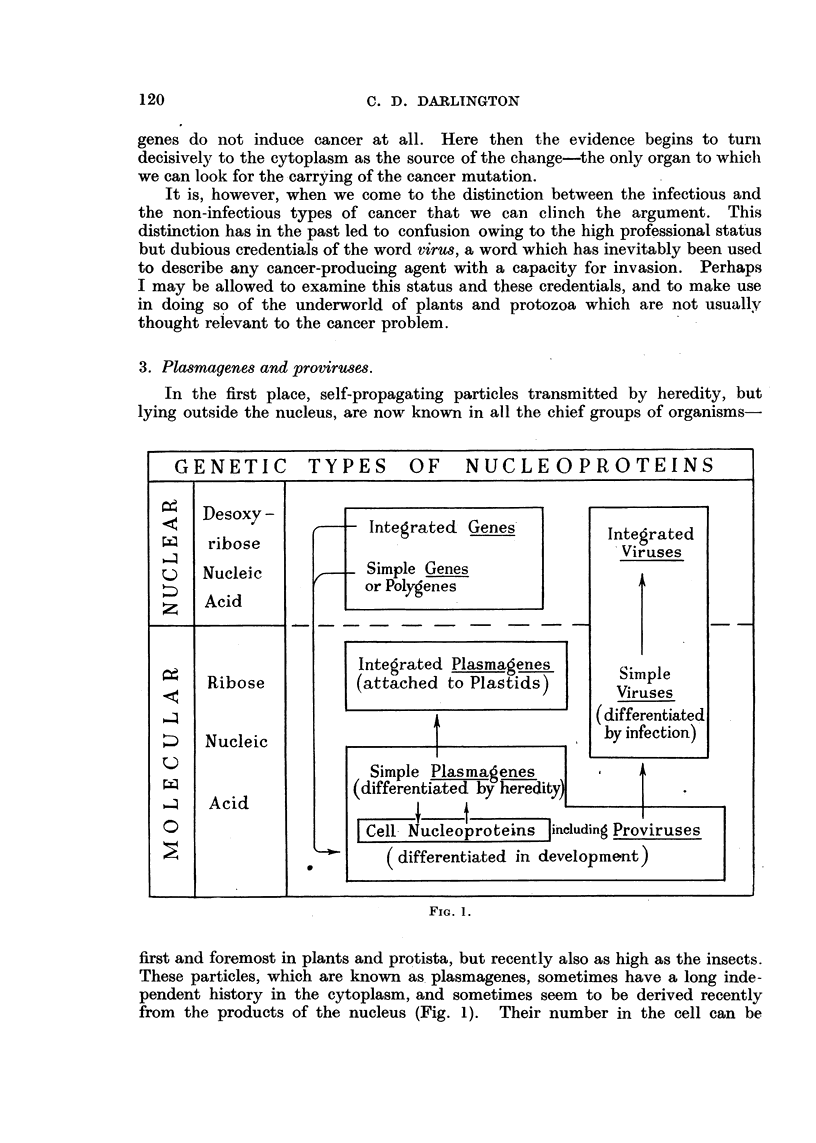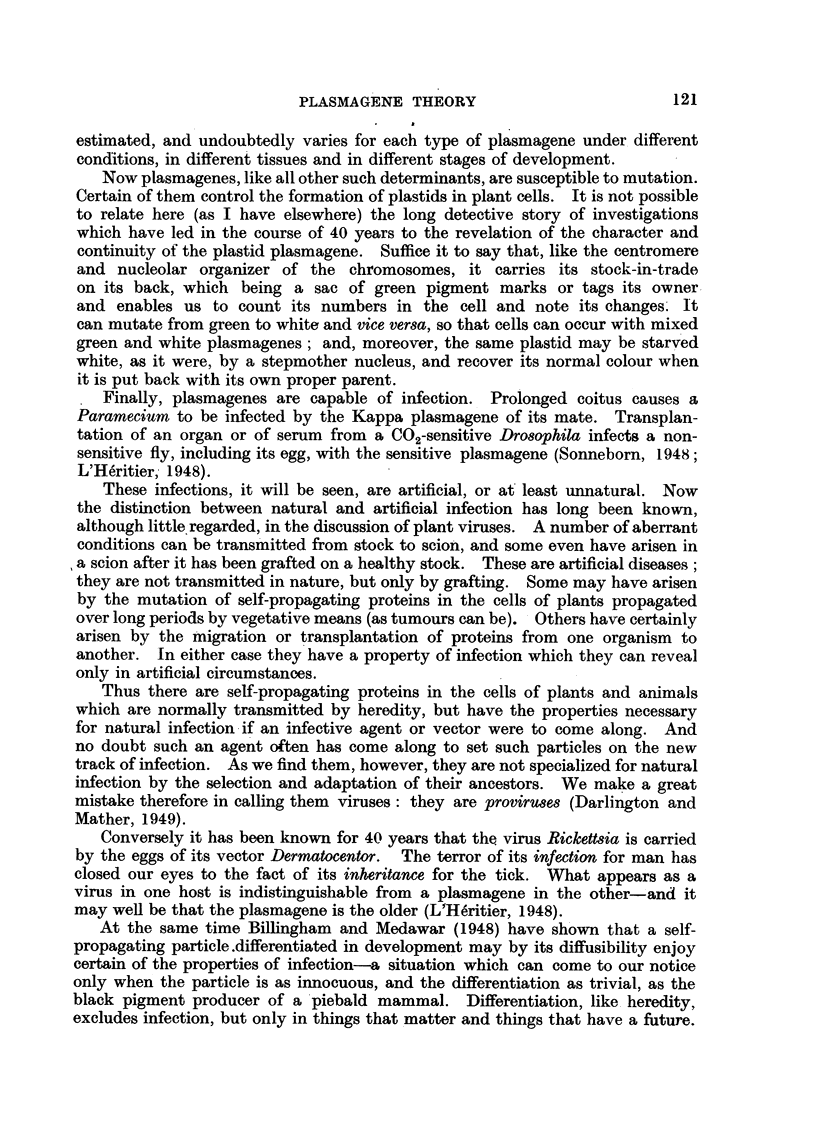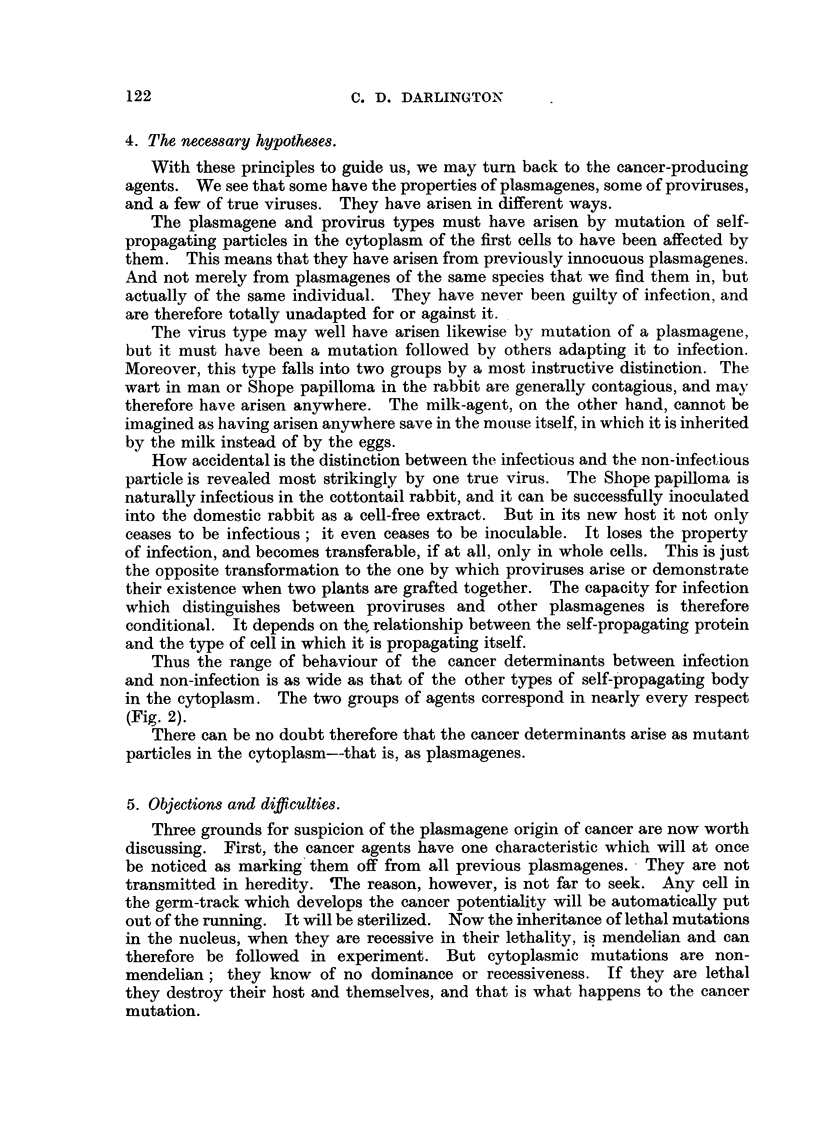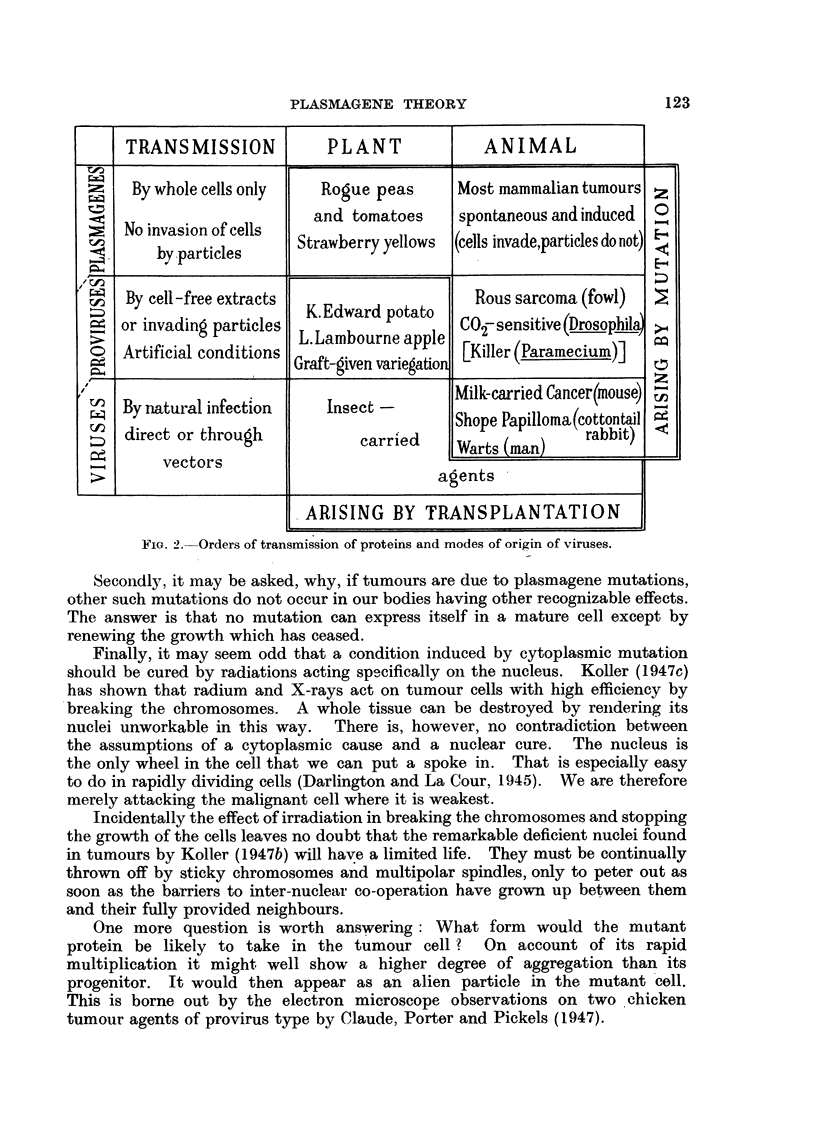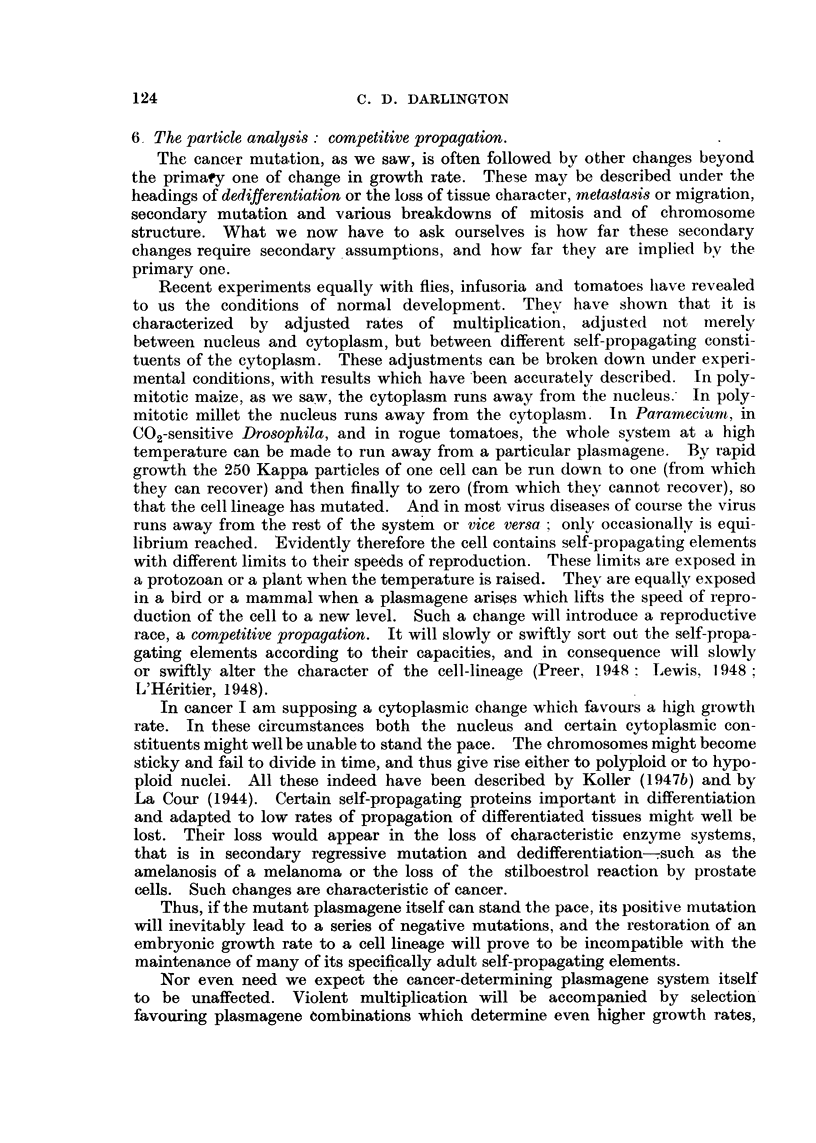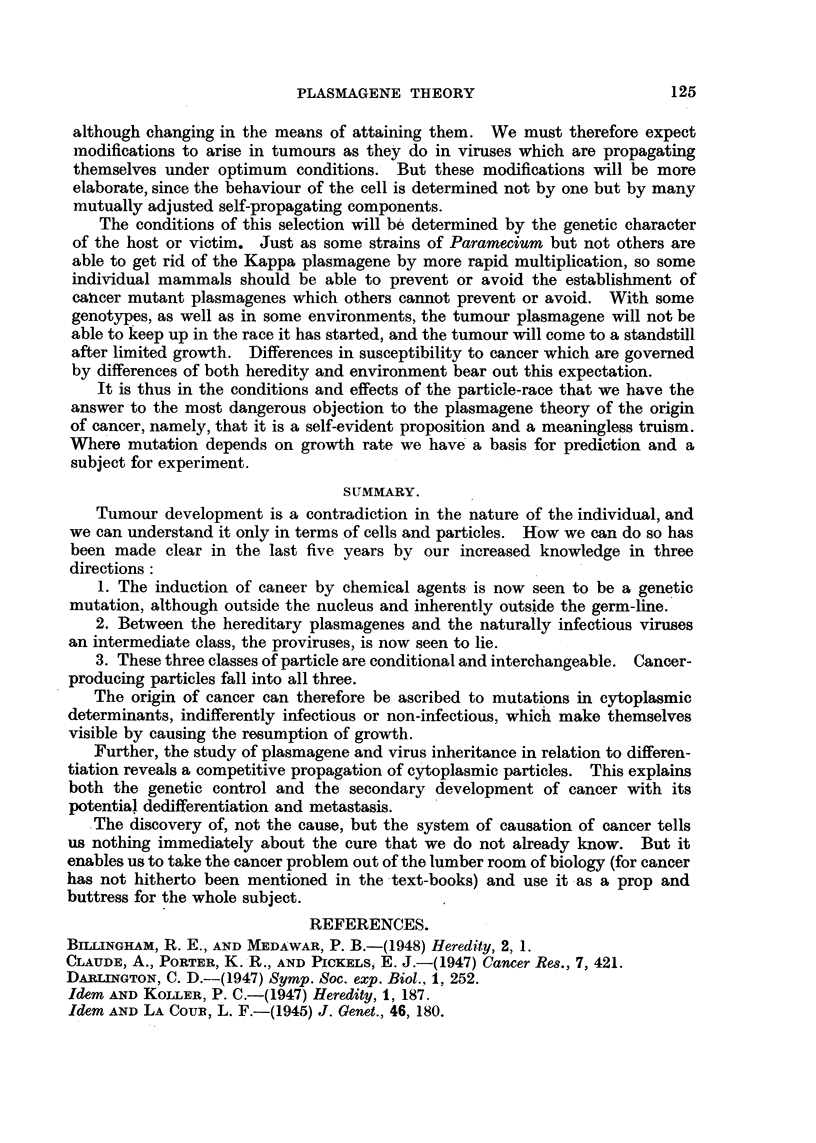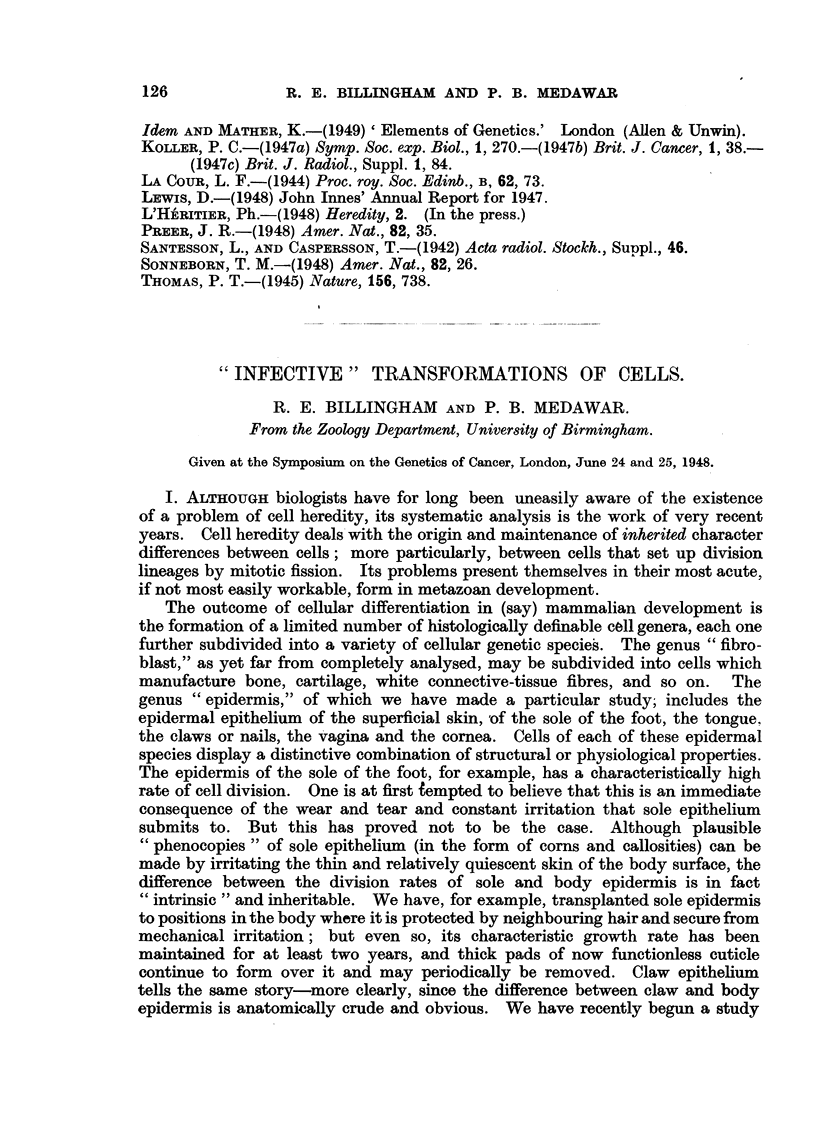# The Plasmagene Theory of the Origin of Cancer

**DOI:** 10.1038/bjc.1948.17

**Published:** 1948-06

**Authors:** C. D. Darlington


### THE PLASMAGENE THEORY OF THE ORIGIN OF CANCER.

C. D. DARLINGTON.

*From the John Innes Horticultural Institution, Merton.*

Given at the Symposium on the Genetics of Cancer, London, June 24 and 25, 1948.

### 1. *The cell analysis.*

Cancer stands at what might be the meeting-place, but what is in fact the no man's land, between the three disciplines of heredity, development, and infection. Its very existence leads to a contradiction between our fundamental notions in these three fields. For this reason we have usually shrunk from asking the crucial questions it puts to us, questions which were indeed, until now, too fundamental to be answered. I must therefore ask those who wish to follow me to be prepared to surrender certain established habits of reasoning, at least for the duration of the journey.

The first problem of cancer is to discover the general principle of its origin, whether in terms of an agent or a process. We are probably all agreed that tumours, although usually, or perhaps always, preceded by abnormal local conditions, arise by a sudden change. This change consists in an increase in the rate of growth of a cell or a group of cells. In this way there arises a new cell-lineage distinct from its antecedents, and one which can propagate its new property irreversibly and even indefinitely. It has the character of a genetic change, a somatic mutation. Secondary changes may follow establishing subsidiary cell-lineages, some involving further increases of growth-rate and dedifferentiation, and all surviving and multiplying subject to natural selection. To these secondary changes I shall return later.

The chemical conditions arising in the changed tissue have been examined by Santesson and Caspersson (1942), while the processes of its cell division have been described by Koller (1947*a*) for epithelial tumours and by La Cour (1944) for pernicious anaemia, which is an analogous condition depending on the enhanced multiplication of the red blood precursor cells of the bone marrow. The conditions and processes fit together. An excess of ribose nucleic acid in the cytoplasm seems to determine more rapid protein production, and more rapid division of cells and nuclei. The rapid nuclear division, as usual in ordinary tissues, reveals chromosomes overcharged with desoxyribose nucleic acid and often improperly co-ordinated with the spindle, so that polyploid as well as deficient cells (with as few as 32 chromosomes) are formed and breakage of chromosomes is also frequent. These errors are no doubt aggravated by the accumulation of lactic acid which arises from the deteriorating conditions of respiration (Thomas, 1945).

The development of unbalanced nuclei in tumours is without precedent in any living tissue. It implies a relaxation of detailed control in the nucleus which is also without precedent. And this in turn argues that the nucleus is not itself directly responsible for what is going on.

Similar behaviour to that of cancer-cells has been observed to arise in plants from the action of particular chromosomes or of nuclear genes. A single polymitotic gene in maize and extra heterochromatic chromosomes in millet both cause rapid and unwanted nuclear divisions in the pollen grain. In maize up to four extra mitoses take place before the chromosomes have had time to divide immediately after meiosis. The nucleus is forced into division, and the unsplit chromosomes are scattered on a spindle which merely disperses them into a number of deficient nuclei, each of which is again compelled to divide before it is ready, and in this way the whole pollen grain is brought to ruin. In millet the mitoses (also up to the number of four) do not occur before the chromosomes are split, but the whole pollen grain is none the less consumed and killed in the end by the production of redundant nuclei. The pollen grain is thus, in a sense, an encapsulated tumour (Darlington, 1947).

Comparison of these cases of polymitosis in plants with animal tumours enables us to clear our minds on one point. Polymitosis is not due to a somatic mutation. It is a property of the whole organism. It affects every cell of a particular type in the body. And it acts as soon as that cell arises. The spontaneous tumour on the other hand arises at random both in time and space. It does not affect all cells of one type, but only one cell, nor is it the first cell of a particular normal type to arise which is affected. We are therefore confirmed in regarding the spontaneous tumour as due to a genetic change.

This conclusion removes the contradiction between development on one side and heredity and infection on the other by withdrawing to a lower level of analysis. The organism is now seen from the cell point of view as a vegetatively propagating colony. The cell is the individual having its own hereditary lineage. So long as the normal course of development is followed it is assumed to propagate itself without change. When a cell in such a colony diverges it does so as a result of a process or agency which may fall into two possible categories: mutation or infection.

### 2. *Mutation and infection.*

Now, when we consider the range of types and conditions of cancer we find that a multiplicity of organisms, tissues and agents is concerned. In this range of variation two differences are significant for our present purpose: that between spontaneous and induced, and that between infectious and non-infectious, cancer.

Cancer, indistinguishable from the natural kinds and in a great variety of kinds, can be produced by chemical agents, the carcinogens. The experimental evidence bears out the *prima facie* case for mutation. How far does it enable us to distinguish between nucleus and cytoplasm as the seat of the mutation? In general the most efficient carcinogens (such as the hydrocarbons) and the most efficient agents of nuclear mutation (such as mustard gas) do not coincide. Carcinogens do not damage the nucleus in proportion to their effect as carcinogens (Darlington and Koller, 1947). Thus the nucleus again seems to be excluded, just as it is by the direct evidence. But the evidence of X-ray effects is a more serious objection to a nuclear origin. X-ray damage leads to the development of cancer only when the dose has been so heavy as to damage the cytoplasm, and only then after prolonged delay. Light doses which have a proportionate effect on the breakage of the chromosomes and the mutation of the genes do not induce cancer at all. Here then the evidence begins to turn decisively to the cytoplasm as the source of the change—the only organ to which we can look for the carrying of the cancer mutation.

It is, however, when we come to the distinction between the infectious and the non-infectious types of cancer that we can clinch the argument. This distinction has in the past led to confusion owing to the high professional status but dubious credentials of the word *virus*, a word which has inevitably been used to describe any cancer-producing agent with a capacity for invasion. Perhaps I may be allowed to examine this status and these credentials, and to make use in doing so of the underworld of plants and protozoa which are not usually thought relevant to the cancer problem.

3. *Plasmagenes and proviruses.*

In the first place, self-propagating particles transmitted by heredity, but lying outside the nucleus, are now known in all the chief groups of organisms—

Fig. 1.

first and foremost in plants and protista, but recently also as high as the insects. These particles, which are known as plasmagenes, sometimes have a long independent history in the cytoplasm, and sometimes seem to be derived recently from the products of the nucleus (Fig. 1). Their number in the cell can be estimated, and undoubtedly varies for each type of plasmagene under different conditions, in different tissues and in different stages of development.

Now plasmagenes, like all other such determinants, are susceptible to mutation. Certain of them control the formation of plastids in plant cells. It is not possible to relate here (as I have elsewhere) the long detective story of investigations which have led in the course of 40 years to the revelation of the character and continuity of the plastid plasmagene. Suffice it to say that, like the centromere and nucleolar organizer of the chromosomes, it carries its stock-in-trade on its back, which being a sac of green pigment marks or tags its owner and enables us to count its numbers in the cell and note its changes. It can mutate from green to white and *vice versa*, so that cells can occur with mixed green and white plasmagenes ; and, moreover, the same plastid may be starved white, as it were, by a stepmother nucleus, and recover its normal colour when it is put back with its own proper parent.

Finally, plasmagenes are capable of infection. Prolonged coitus causes a *Paramecium* to be infected by the Kappa plasmagene of its mate. Transplantation of an organ or of serum from a $CO_2$-sensitive *Drosophila* infects a non-sensitive fly, including its egg, with the sensitive plasmagene (Sonneborn, 1948; L'Héritier, 1948).

These infections, it will be seen, are artificial, or at least unnatural. Now the distinction between natural and artificial infection has long been known, although little regarded, in the discussion of plant viruses. A number of aberrant conditions can be transmitted from stock to scion, and some even have arisen in a scion after it has been grafted on a healthy stock. These are artificial diseases ; they are not transmitted in nature, but only by grafting. Some may have arisen by the mutation of self-propagating proteins in the cells of plants propagated over long periods by vegetative means (as tumours can be). Others have certainly arisen by the migration or transplantation of proteins from one organism to another. In either case they have a property of infection which they can reveal only in artificial circumstances.

Thus there are self-propagating proteins in the cells of plants and animals which are normally transmitted by heredity, but have the properties necessary for natural infection if an infective agent or vector were to come along. And no doubt such an agent often has come along to set such particles on the new track of infection. As we find them, however, they are not specialized for natural infection by the selection and adaptation of their ancestors. We make a great mistake therefore in calling them viruses : they are *proviruses* (Darlington and Mather, 1949).

Conversely it has been known for 40 years that the virus *Rickettsia* is carried by the eggs of its vector *Dermatocentor*. The terror of its *infection* for man has closed our eyes to the fact of its *inheritance* for the tick. What appears as a virus in one host is indistinguishable from a plasmagene in the other—and it may well be that the plasmagene is the older (L'Héritier, 1948).

At the same time Billingham and Medawar (1948) have shown that a self-propagating particle differentiated in development may by its diffusibility enjoy certain of the properties of infection—a situation which can come to our notice only when the particle is as innocuous, and the differentiation as trivial, as the black pigment producer of a piebald mammal. Differentiation, like heredity, excludes infection, but only in things that matter and things that have a future.

4. *The necessary hypotheses.*

With these principles to guide us, we may turn back to the cancer-producing agents. We see that some have the properties of plasmagenes, some of proviruses, and a few of true viruses. They have arisen in different ways.

The plasmagene and provirus types must have arisen by mutation of self-propagating particles in the cytoplasm of the first cells to have been affected by them. This means that they have arisen from previously innocuous plasmagenes. And not merely from plasmagenes of the same species that we find them in, but actually of the same individual. They have never been guilty of infection, and are therefore totally unadapted for or against it.

The virus type may well have arisen likewise by mutation of a plasmagene, but it must have been a mutation followed by others adapting it to infection. Moreover, this type falls into two groups by a most instructive distinction. The wart in man or Shope papilloma in the rabbit are generally contagious, and may therefore have arisen anywhere. The milk-agent, on the other hand, cannot be imagined as having arisen anywhere save in the mouse itself, in which it is inherited by the milk instead of by the eggs.

How accidental is the distinction between the infectious and the non-infectious particle is revealed most strikingly by one true virus. The Shope papilloma is naturally infectious in the cottontail rabbit, and it can be successfully inoculated into the domestic rabbit as a cell-free extract. But in its new host it not only ceases to be infectious ; it even ceases to be inoculable. It loses the property of infection, and becomes transferable, if at all, only in whole cells. This is just the opposite transformation to the one by which proviruses arise or demonstrate their existence when two plants are grafted together. The capacity for infection which distinguishes between proviruses and other plasmagenes is therefore conditional. It depends on the relationship between the self-propagating protein and the type of cell in which it is propagating itself.

Thus the range of behaviour of the cancer determinants between infection and non-infection is as wide as that of the other types of self-propagating body in the cytoplasm. The two groups of agents correspond in nearly every respect (Fig. 2).

There can be no doubt therefore that the cancer determinants arise as mutant particles in the cytoplasm—that is, as plasmagenes.

5. *Objections and difficulties.*

Three grounds for suspicion of the plasmagene origin of cancer are now worth discussing. First, the cancer agents have one characteristic which will at once be noticed as marking them off from all previous plasmagenes. They are not transmitted in heredity. The reason, however, is not far to seek. Any cell in the germ-track which develops the cancer potentiality will be automatically put out of the running. It will be sterilized. Now the inheritance of lethal mutations in the nucleus, when they are recessive in their lethality, is mendelian and can therefore be followed in experiment. But cytoplasmic mutations are non-mendelian ; they know of no dominance or recessiveness. If they are lethal they destroy their host and themselves, and that is what happens to the cancer mutation.

| | TRANSMISSION | PLANT | ANIMAL |
|---|---|---|---|
| PLASMAGENES | By whole cells only<br>No invasion of cells by particles | Rogue peas and tomatoes<br>Strawberry yellows | Most mammalian tumours spontaneous and induced (cells invade, particles do not) |
| PROVIRUSES | By cell-free extracts or invading particles<br>Artificial conditions | K. Edward potato<br>L. Lambourne apple<br>Graft-given variegation | Rous sarcoma (fowl)<br>$CO_2$-sensitive (*Drosophila*)<br>[Killer (*Paramecium*)] |
| VIRUSES | By natural infection direct or through vectors | Insect — carried agents | Milk-carried Cancer (mouse)<br>Shope Papilloma (cottontail rabbit)<br>Warts (man) |

ARISING BY MUTATION

ARISING BY TRANSPLANTATION

FIG. 2.—Orders of transmission of proteins and modes of origin of viruses.

Secondly, it may be asked, why, if tumours are due to plasmagene mutations, other such mutations do not occur in our bodies having other recognizable effects. The answer is that no mutation can express itself in a mature cell except by renewing the growth which has ceased.

Finally, it may seem odd that a condition induced by cytoplasmic mutation should be cured by radiations acting specifically on the nucleus. Koller (1947*c*) has shown that radium and X-rays act on tumour cells with high efficiency by breaking the chromosomes. A whole tissue can be destroyed by rendering its nuclei unworkable in this way. There is, however, no contradiction between the assumptions of a cytoplasmic cause and a nuclear cure. The nucleus is the only wheel in the cell that we can put a spoke in. That is especially easy to do in rapidly dividing cells (Darlington and La Cour, 1945). We are therefore merely attacking the malignant cell where it is weakest.

Incidentally the effect of irradiation in breaking the chromosomes and stopping the growth of the cells leaves no doubt that the remarkable deficient nuclei found in tumours by Koller (1947*b*) will have a limited life. They must be continually thrown off by sticky chromosomes and multipolar spindles, only to peter out as soon as the barriers to inter-nuclear co-operation have grown up between them and their fully provided neighbours.

One more question is worth answering: What form would the mutant protein be likely to take in the tumour cell? On account of its rapid multiplication it might well show a higher degree of aggregation than its progenitor. It would then appear as an alien particle in the mutant cell. This is borne out by the electron microscope observations on two chicken tumour agents of provirus type by Claude, Porter and Pickels (1947).

6. *The particle analysis : competitive propagation.*

The cancer mutation, as we saw, is often followed by other changes beyond the primary one of change in growth rate. These may be described under the headings of *dedifferentiation* or the loss of tissue character, *metastasis* or migration, secondary mutation and various breakdowns of mitosis and of chromosome structure. What we now have to ask ourselves is how far these secondary changes require secondary assumptions, and how far they are implied by the primary one.

Recent experiments equally with flies, infusoria and tomatoes have revealed to us the conditions of normal development. They have shown that it is characterized by adjusted rates of multiplication, adjusted not merely between nucleus and cytoplasm, but between different self-propagating constituents of the cytoplasm. These adjustments can be broken down under experimental conditions, with results which have been accurately described. In polymitotic maize, as we saw, the cytoplasm runs away from the nucleus. In polymitotic millet the nucleus runs away from the cytoplasm. In *Paramecium*, in $CO_2$-sensitive *Drosophila*, and in rogue tomatoes, the whole system at a high temperature can be made to run away from a particular plasmagene. By rapid growth the 250 Kappa particles of one cell can be run down to one (from which they can recover) and then finally to zero (from which they cannot recover), so that the cell lineage has mutated. And in most virus diseases of course the virus runs away from the rest of the system or *vice versa* ; only occasionally is equilibrium reached. Evidently therefore the cell contains self-propagating elements with different limits to their speeds of reproduction. These limits are exposed in a protozoan or a plant when the temperature is raised. They are equally exposed in a bird or a mammal when a plasmagene arises which lifts the speed of reproduction of the cell to a new level. Such a change will introduce a reproductive race, a *competitive propagation.* It will slowly or swiftly sort out the self-propagating elements according to their capacities, and in consequence will slowly or swiftly alter the character of the cell-lineage (Preer, 1948 ; Lewis, 1948 ; L'Héritier, 1948).

In cancer I am supposing a cytoplasmic change which favours a high growth rate. In these circumstances both the nucleus and certain cytoplasmic constituents might well be unable to stand the pace. The chromosomes might become sticky and fail to divide in time, and thus give rise either to polyploid or to hypoploid nuclei. All these indeed have been described by Koller (1947*b*) and by La Cour (1944). Certain self-propagating proteins important in differentiation and adapted to low rates of propagation of differentiated tissues might well be lost. Their loss would appear in the loss of characteristic enzyme systems, that is in secondary regressive mutation and dedifferentiation—such as the amelanosis of a melanoma or the loss of the stilboestrol reaction by prostate cells. Such changes are characteristic of cancer.

Thus, if the mutant plasmagene itself can stand the pace, its positive mutation will inevitably lead to a series of negative mutations, and the restoration of an embryonic growth rate to a cell lineage will prove to be incompatible with the maintenance of many of its specifically adult self-propagating elements.

Nor even need we expect the cancer-determining plasmagene system itself to be unaffected. Violent multiplication will be accompanied by selection favouring plasmagene combinations which determine even higher growth rates, although changing in the means of attaining them. We must therefore expect modifications to arise in tumours as they do in viruses which are propagating themselves under optimum conditions. But these modifications will be more elaborate, since the behaviour of the cell is determined not by one but by many mutually adjusted self-propagating components.

The conditions of this selection will be determined by the genetic character of the host or victim. Just as some strains of *Paramecium* but not others are able to get rid of the Kappa plasmagene by more rapid multiplication, so some individual mammals should be able to prevent or avoid the establishment of cancer mutant plasmagenes which others cannot prevent or avoid. With some genotypes, as well as in some environments, the tumour plasmagene will not be able to keep up in the race it has started, and the tumour will come to a standstill after limited growth. Differences in susceptibility to cancer which are governed by differences of both heredity and environment bear out this expectation.

It is thus in the conditions and effects of the particle-race that we have the answer to the most dangerous objection to the plasmagene theory of the origin of cancer, namely, that it is a self-evident proposition and a meaningless truism. Where mutation depends on growth rate we have a basis for prediction and a subject for experiment.

### SUMMARY.

Tumour development is a contradiction in the nature of the individual, and we can understand it only in terms of cells and particles. How we can do so has been made clear in the last five years by our increased knowledge in three directions :

1. The induction of cancer by chemical agents is now seen to be a genetic mutation, although outside the nucleus and inherently outside the germ-line.
2. Between the hereditary plasmagenes and the naturally infectious viruses an intermediate class, the proviruses, is now seen to lie.
3. These three classes of particle are conditional and interchangeable. Cancer-producing particles fall into all three.

The origin of cancer can therefore be ascribed to mutations in cytoplasmic determinants, indifferently infectious or non-infectious, which make themselves visible by causing the resumption of growth.

Further, the study of plasmagene and virus inheritance in relation to differentiation reveals a competitive propagation of cytoplasmic particles. This explains both the genetic control and the secondary development of cancer with its potential dedifferentiation and metastasis.

The discovery of, not the cause, but the system of causation of cancer tells us nothing immediately about the cure that we do not already know. But it enables us to take the cancer problem out of the lumber room of biology (for cancer has not hitherto been mentioned in the text-books) and use it as a prop and buttress for the whole subject.

### REFERENCES.

BILLINGHAM, R. E., AND MEDAWAR, P. B.—(1948) *Heredity*, 2, 1.
CLAUDE, A., PORTER, K. R., AND PICKELS, E. J.—(1947) *Cancer Res.*, 7, 421.
DARLINGTON, C. D.—(1947) *Symp. Soc. exp. Biol.*, 1, 252.
*Idem* AND KOLLER, P. C.—(1947) *Heredity*, 1, 187.
*Idem* AND LA COUR, L. F.—(1945) *J. Genet.*, **46**, 180.

*Idem* AND MATHER, K.—(1949) ' Elements of Genetics.' London (Allen & Unwin).
KOLLER, P. C.—(1947*a*) *Symp. Soc. exp. Biol.*, **1**, 270.—(1947*b*) *Brit. J. Cancer*, **1**, 38.—(1947*c*) *Brit. J. Radiol.*, Suppl. **1**, 84.
LA COUR, L. F.—(1944) *Proc. roy. Soc. Edinb.*, B, **62**, 73.
LEWIS, D.—(1948) John Innes' Annual Report for 1947.
L'HÉRITIER, Ph.—(1948) *Heredity*, **2**. (In the press.)
PREER, J. R.—(1948) *Amer. Nat.*, **82**, 35.
SANTESSON, L., AND CASPERSSON, T.—(1942) *Acta radiol. Stockh.*, Suppl., **46**.
SONNEBORN, T. M.—(1948) *Amer. Nat.*, **82**, 26.
THOMAS, P. T.—(1945) *Nature*, **156**, 738.